# The Rootstock Regulates Microbiome Diversity in Root and Rhizosphere Compartments of *Vitis vinifera* Cultivar Lambrusco

**DOI:** 10.3389/fmicb.2018.02240

**Published:** 2018-09-26

**Authors:** Federica D’Amico, Marco Candela, Silvia Turroni, Elena Biagi, Patrizia Brigidi, Alessia Bega, Davide Vancini, Simone Rampelli

**Affiliations:** ^1^Unit of Microbial Ecology of Health, Department of Pharmacy and Biotechnology, University of Bologna, Bologna, Italy; ^2^Istituto d’Istruzione Superiore Ignazio Calvi, Modena, Italy

**Keywords:** microbiota, microbiome, soil-plant interface, grapevine, rootstock

## Abstract

Plants belonging to *Vitis vinifera* varieties are usually grafted on different rootstocks to enhance the plant defenses against pathogens and increase productivity under harsh environmental conditions. Particularly, in Emilia-Romagna region (Italy), *Vitis vinifera* cultivar Lambrusco can be grafted on a hybrid of *V. berlandieri* × *V. riparia* (5BB) or *V. berlandieri* × *V. rupestris* (1103P). However, the latter shows potassium absorption problems, with a consequent reduction in grapevine production. Since it has recently been demonstrated that the rootstock has the potential to select for different microorganisms at the root-soil interface, here we hypothesized that the potassium deficiency of 1103P could be partially accounted for by the peculiarities of the rootstock microbiome. We thus employed 16S rRNA sequencing to compare root and rhizosphere microbiomes in plants of *V. vinifera* cultivar Lambrusco grafted on the two aforementioned rootstocks. According to our findings, 1103P shows a reduced diversity in root and rhizosphere microbiomes, including members of potassium-solubilizing microorganisms, possibly explaining the inadequate potassium absorption of this hybrid. Besides confirming the importance of the rootstock as a determinant of the composition of plant microbiomes, our data indicate the relevance of rootstock-selected microbiomes as possible regulators of potassium absorption by *V. vinifera*.

## Introduction

Multicellular organisms can no longer be considered individuals by the classical definitions of the term. Plants, as well as animals, are indeed holobionts, defined as meta-organisms including the host and its associated microbial communities, which are collectively referred to as the microbiota (Margulis, 1991; Hardoim et al., 2008; Ryan et al., 2008; Rosenberg and Zilber-Rosenberg, 2016). In particular, the plant microbiota includes the microorganisms that populate the thin layer of soil adhering to the roots (named as rhizosphere), those that inhabit the root surface (rhizoplane), and those colonizing the root interior as well as other inner tissues of the plant (endosphere) (Kloepper et al., 1980; Kowalchuk et al., 2002; Bulgarelli et al., 2013).

The grape has a long winemaking history with archeological evidence indicating that also Etruscans and Romans cultivated the grapevine (Robinson, 1986). In Italy, the grapevine cultivation has a strong economic importance due to the valuable quality of the wine. Therefore, it is important to preserve the plant growth from any injury, in particular from the *Phylloxera* infection. Also for this reason, all *Vitis vinifera* varieties are cultivated as scions and grafted on rootstocks of other *Vitis* species (Whiting, 2003). In addition to the importance of rootstocks in enhancing the plant defenses against pathogens (Whiting, 2003; Wallis et al., 2013), they are strategic in increasing *V. vinifera* productivity under harsh environmental conditions, while simultaneously limiting agricultural inputs (irrigation, fertilizer, and pesticides) (Zarraonaindia et al., 2015; Warschefsky et al., 2016). Moreover, rootstocks are important to prevent problems due to soil conditions, such as salinity or poor mineral nutrition (Habran et al., 2016).

Perhaps not surprisingly, the rootstock choice plays a critical role in the winemaking process. A paradigmatic example of this phenomenon is the production of Lambrusco wine. In Emilia-Romagna, the Italian region leading Lambrusco’s production globally^1^, a dichotomy can be observed: grapevines are grafted on rootstock hybrids of either *V. berlandieri* × *V. rupestris* or *V. berlandieri* × *V. riparia*. The two hybrids show different characteristics and performance depending on the quality of the soil. Both are suitable for a clay soil, however, *V. berlandieri* × *V. rupestris* shows less absorption of potassium (K) that is the third most important nutrient of the plant, after nitrogen and phosphorus (Wolpert et al., 2005). When the defects in K absorption are such that the vigor of the grapevine can no longer be maintained, this deficiency leads to inhibition of photosynthesis and to sucrose being “trapped” in the leaves, which adversely affects the yield, the fruit ripening and the concentration of the sugar in the grapes. Moreover, an inadequate K absorption can lead to poor development of roots, slow growth and increased susceptibility to diseases and pest (Keller, 2010).

It is well known that rhizospheric microorganisms, particularly K-solubilizing microorganisms (KSMs), are able to solubilize insoluble minerals into soluble forms useful for plant metabolism (Meena et al., 2014; Etesami et al., 2017). Despite the molecular mechanisms by which microorganisms regulate the K solubilization are still not clear, a wide range of KSMs have been characterized, such as *Bacillus mucilaginosus*, *Bacillus edaphicus* (Meena et al., 2014), *Burkholderia*, *Aminobacter*, and *Sphingomonas* (Uroz et al., 2007; Zhang and Kong, 2014), as well as microorganisms belonging to *Rhizobiaceae*, *Cytophagaceae*, and *Micrococcaceae* families (Diep and Hieu, 2013; Zarjani et al., 2013; Zhang and Kong, 2014). Since the rootstock has recently been reported as a determinant of the bacterial composition in the root system compartments of *V. vinifera* (Liu et al., 2018; Marasco et al., 2018), we hypothesized that the different performance of *V. berlandieri* × *V. rupestris* and *V. berlandieri* × *V. riparia* rootstocks in K absorption is attributable to compositional differences in the microbiomes from the root compartments. In this scenario, here we characterized the bacterial community of soil, rhizosphere and root endosphere in *V. vinifera* cultivar Lambrusco grafted on the two aforementioned types of rootstock showing high or low performance in K absorption, i.e., *V. berlandieri* × *V. riparia* and *V. berlandieri* × *V. rupestris*, respectively. In order to avoid biases related to weather conditions and soil type, all plants were grown in the same field. Our data provides some glimpses on the microbial *terroir* of grapevines, highlighting the evolution of a specific relationship between rootstock, microbiota and plant physiology, and suggesting the possibility of improving grapevine physiology by complementing the rootstock microbiome through the supplementation of probiotic microbes to the soil.

## Materials and Methods

### Experimental Design and Sampling

Grafted Lambrusco grapevine plants (*Vitis vinifera* cultivar Lambrusco) were sampled at Istituto d’Istruzione Superiore (IIS) Ignazio Calvi (44.839 N/11.285 E, Finale Emilia, Modena, Italy) in November 2016. Specifically, two different rootstocks were selected: *V. berlandieri* × *V. rupestris* PAULSEN 1103 (1103P) and *V. berlandieri* × *V. riparia* KOBER 5BB (5BB) (**Table 1**). Eight 1103P and seven 5BB grapevine plants were randomly sampled from three rows of the same vineyard field. Physical and chemical properties of the soil from an unplanted area were determined (**Supplementary Table S1**).

**Table 1:** Characteristics of the two rootstocks used in the present study.

Rootstock	Parentage	Vigor	Drought resistance	Lime tolerance (%)†	Salt resistance	Wet feet‡	Soil preference§
**1103P**	*V. berlandieri* × *V. rupestris*	H	H	17	M	H	Adapted to drought, saline soils
**5BB**	*V. berlandieri* × *V. riparia*	M	L/M	20	L/M	Var	Moist clay


*L = low; M = medium; H = high; Var = variable. †Tolerance to lime-induced chlorosis (percent by weight of finely divided calcium carbonate in soil that can be tolerated by the rootstock). ‡Tolerance to excessive moisture caused by poor soil drainage. §Actual performance characteristics on specific soils and scions may vary. Sources: Adapted from Lambert et al. (2008).*

For the microbiota analysis, for each plant, three compartments were analyzed: soil, rhizosphere and root endosphere for a total of 45 samples. The soil compartment was sampled next to each plant after removing the top 0.5–1 cm of soil. Roots were manually removed from the soil using sterile gloves and processed to separate the rhizosphere from the root endosphere, as previously described in Bulgarelli et al. (2012). Briefly, 3 cm of root segments, including the root tips, were dissected with a sterile scalpel, to standardize sampling. The root sections were collected in 15 mL Falcon tubes containing 2.5 mL of modified PBS buffer (130 mM NaCl, 7 mM Na_2_HPO_4_, 3 mM NaH_2_PO_4_, pH 7.0, 0.02% Silwet L-77) and washed on a shaking platform at 180 rpm for 20 min. The rhizosphere compartment was defined as the pellet resulting from the centrifugation of the washing buffer at 1.500 × *g* for 20 min. After a second washing step under the same conditions, roots were transferred to another Falcon tube with 2.5 mL of modified PBS buffer and subjected to 10 cycles of sonication consisting of 30-s pulses at 160 W with 30-s breaks in an ultrasonic bath (Branson 1800, Branson Ultrasonic Corporation, Danbury, CT, United States). Washed and sonicated roots were grinded with mortar and pestle, and defined as the root endosphere compartment. All samples were stored at -80°C until DNA extraction.

### DNA Extraction and Sequencing

DNA was extracted from all the 45 samples from soil, rhizosphere and root endosphere compartments using the DNeasy PowerSoil Kit (QIAGEN, Hilden, Germany) as per the manufacturer’s instructions, except for the homogenization step that was performed in a FastPrep instrument (MP Biomedicals, Irvine, CA, United States) by three 1-min steps at 5.5 movements per sec. The V3–V4 hypervariable region of the 16S rRNA gene was amplified using the 341F and 785R primers with added Illumina adapter overhang sequences as previously reported (Turroni et al., 2016). Briefly, the thermal cycle consisted of initial denaturation at 95°C for 3 min, 25 cycles of denaturation at 95°C for 30 s, annealing at 55°C for 30 s and extension at 72°C for 30 s, and a final extension step at 72°C for 5 min. PCR reactions were cleaned up with Agencourt AMPure XP magnetic beads (Beckman Coulter, Brea, CA, United States). Indexed libraries were prepared by limited-cycle PCR using Nextera technology. After a further clean-up step as described above, libraries were normalized to 4 nM and pooled. The sample pool was denatured with 0.2 N NaOH and diluted to 6 pM with a 20% PhiX control. Sequencing was performed on Illumina MiSeq platform using a 2 × 250 bp paired end protocol, according to the manufacturer’s instructions (Illumina, San Diego, CA, United States). Sequencing reads were deposited in MG-RAST^2^

### Bioinformatics and Statistical Analysis

Raw sequences were processed using a pipeline combining PANDAseq (Masella et al., 2012) and QIIME (Caporaso et al., 2011) using default parameters. Reads shorter than 350 bp or longer than 500 bp were discarded. Quality-filtered sequences were clustered into OTUs at 97% similarity threshold using UCLUST (Edgar, 2010), and taxonomy was assigned using the RDP classifier and the Greengenes database (May 2013 release). The filtering of chimeric OTUs was performed by using ChimeraSlayer (Haas et al., 2011). All singleton OTUs were discarded. We excluded the Cyanobacteria phylum from all analysis to prevent any problem due to the concomitant detection of plant chloroplasts (mean percentage of reads assigned to chloroplasts in root endosphere samples = 27.25%).

Alpha-diversity was evaluated using three different metrics: Shannon, PD whole tree, and observed OTUs. Weighted and unweighted UniFrac distances were used to perform PCoA. PCoA and bar plots were built using the R packages “Made4” (Culhane et al., 2005) and “Vegan”^3^. Ternary plots were prepared using the R packages “vcd” (Meyer et al., 2017) and “ggtern” (Hamilton, 2017). The R packages “Stats” and “Vegan” were used to perform statistical analysis. In particular, to compare the microbiota structure among different groups for alpha and beta-diversity, we used a Mann-Whitney-Wilcoxon test. Data separation in the PCoA was tested using a permutation test with pseudo-F ratios (function “Adonis” in the “Vegan” package). Tropic groups [i.e., groups of OTUs enriched in the root endosphere (Ro_OTUs), in the rhizosphere (Rh_OTUs) or in both (RR_OTUs)] were defined based on OTU relative abundance values, as previously shown in Bulgarelli et al. (2012). Differentially abundant OTUs between microhabitats were detected using Mann-Whitney-Wilcoxon test. OTU niche differentiation (i.e., the OTU preference for the soil, the root endosphere and/or the rhizosphere) was defined based on the OTU differential representation among microhabitats. KSM-related OTUs were defined based on the literature available on microorganisms known for their K solubilization activity. Significant differences in bacterial relative abundance at different phylogenetic levels between microhabitats and plant groups, were assessed by Mann-Whitney-Wilcoxon test or Kruskal-Wallis test, and corrected for multiple comparisons using the Benjamini-Hochberg method when appropriate. False discovery rate (FDR) <0.05 was considered as statistically significant.

## Results

We worked on *Vitis vinifera* cultivar Lambrusco collected at Istituto d’Istruzione Superiore (IIS) Ignazio Calvi located in Finale Emilia (Modena, Italy). We analyzed two different groups of plants grafted onto different rootstocks: eight plants of *V. vinifera* grafted on the hybrid *V. berlandieri* × *V. rupestris* PAULSEN 1103 (1103P) and seven plants of *V. vinifera* grafted on the hybrid *V. berlandieri* × *V. riparia* KOBER 5BB (5BB). To exclude biases due to different soil characteristics (e.g., pH, moisture and temperature), as well as to a different soil microbiome layout, all plants were from the same vineyard (**Supplementary Table S1**). Although the cultivar was the same, the *V. vinifera* group 1103P showed problems of nutrient absorption, especially of potassium (K), as assessed by visual inspection of foliar symptoms, a standard approach to determine whether nutrient concentration is adequate for plant growth (Wolf, 2008). The leaves of *V. vinifera* 1103P were indeed characterized by widespread red spots, an established consequence of K deficiency (**Supplementary Figure S1**).

### Overall Structure of Bacterial Communities Associated With the Root System of Grafted Grapevine

**FIGURE 1:**
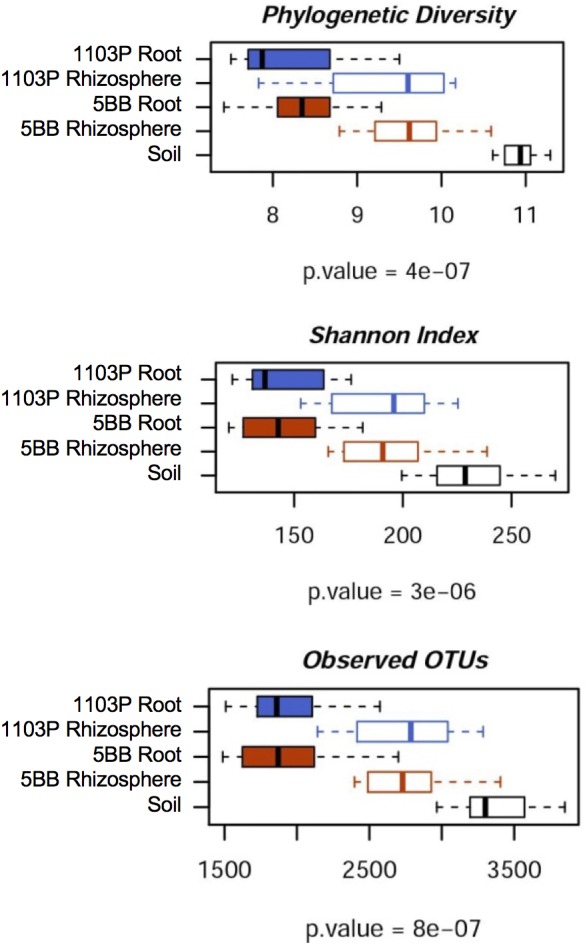
Microbiota biodiversity in soil, rhizosphere, and root compartments for *V. vinifera* cultivar Lambrusco grafted onto different rootstocks. The OTU table at 0.03 similarity threshold was rarefied up to 10,277 reads per sample and analyzed using the following diversity metrics: Faith’s Phylogenetic Diversity (PD whole tree), the Shannon index of biodiversity and observed OTUs. All metrics show greater microbiota diversity in the soil compared to rhizosphere and root compartments, for both *V. vinifera* 1103P and *V. vinifera* 5BB (Kruskal-Wallis test).

**FIGURE 2:**
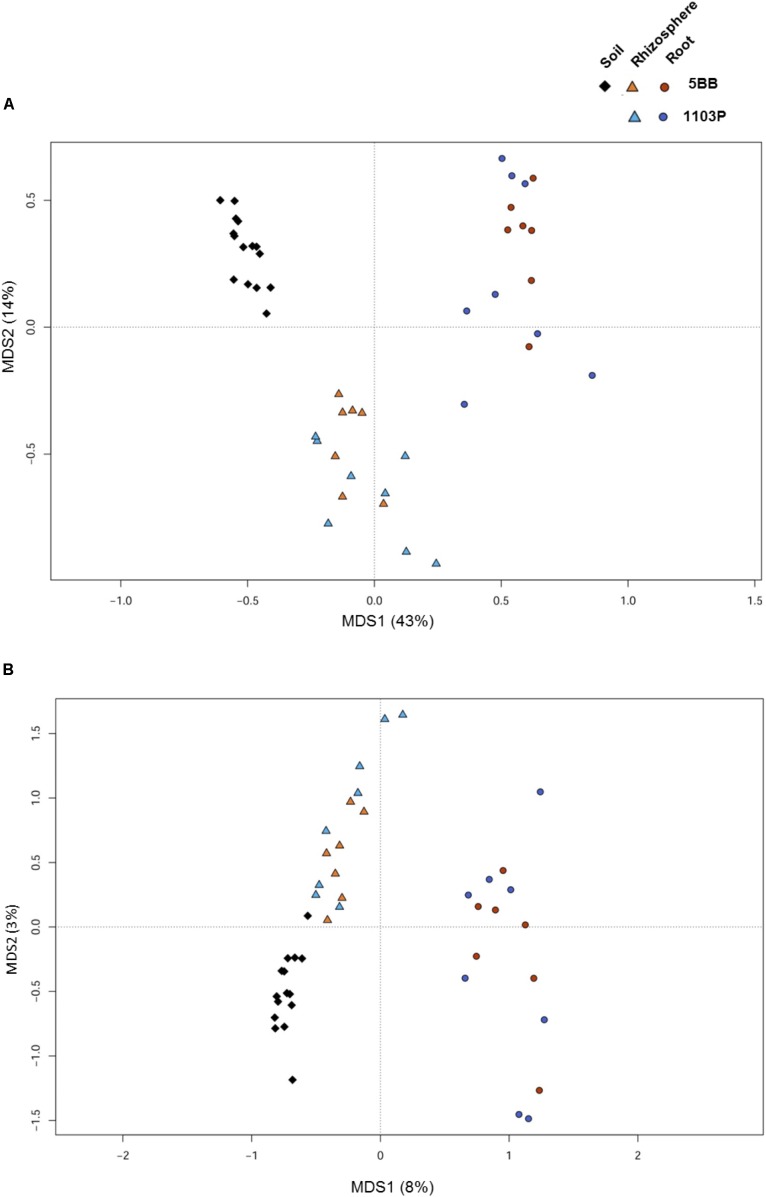
Microbiota community structure in soil, rhizosphere, and root compartments for *V. vinifera* cultivar Lambrusco grafted onto different rootstocks. Weighted **(A)** and unweighted **(B)** UniFrac distance PCoA shows significant segregation among soil (diamonds), rhizosphere (triangles), and root (circles) samples for both *V. vinifera* 1103P and *V. vinifera* 5BB. Permutational multivariate ANOVA based on distance matrices (Adonis), *P* = 0.0001.

**FIGURE 3:**
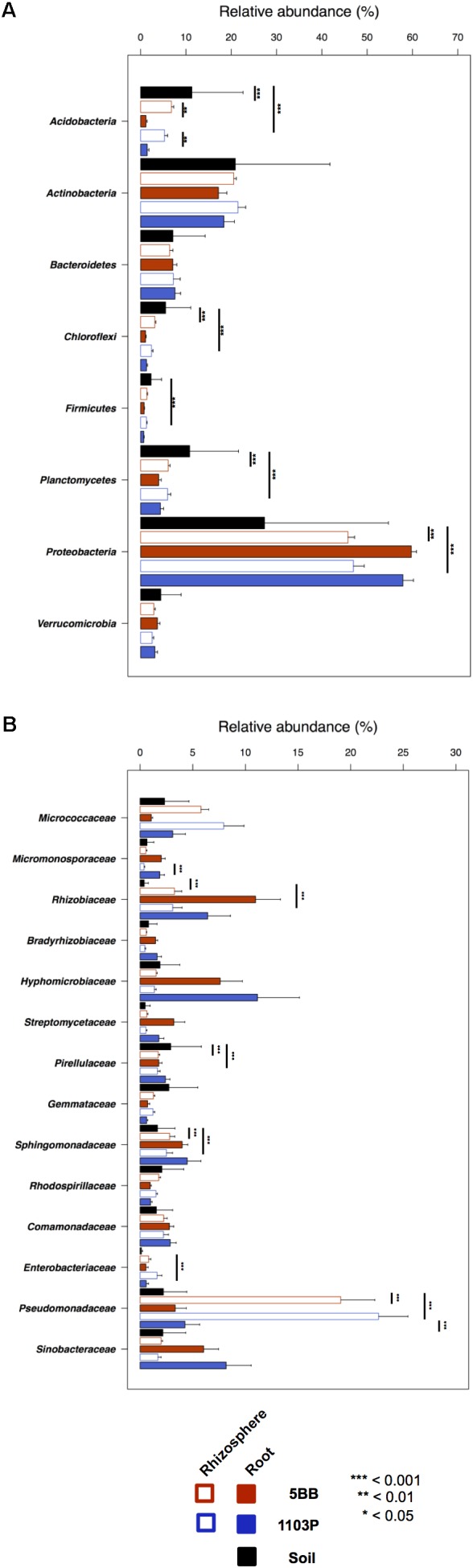
Taxonomic composition of bacterial communities in soil, rhizosphere, and root compartments for *V. vinifera* cultivar Lambrusco grafted onto different rootstocks. Relative abundance (mean ± SEM) of the most abundant phyla **(A)** and families **(B)** in soil, rhizosphere, and root samples for both groups of plants analyzed (*V. vinifera* 1103P and *V. vinifera* 5BB). Stars indicate significant differences [Mann-Whitney-Wilcoxon test, false discovery rate (FDR)-adjustment]. Only taxa with relative abundance >0.5% in at least 15 samples were included.

For each of the 15 plants of *V. vinifera* cultivar Lambrusco collected in this study, we analyzed the root endosphere (hereafter referred to as root), rhizosphere and soil compartments by next-generation sequencing of the V3-V4 hypervariable regions of 16S rDNA. The sequencing generated 5,444,065 high-quality reads (mean, 120,979; range, 34,386–2,683,522) that were clustered into 22,570 operational taxonomic units (OTUs) at 97% identity. We used different metrics to calculate alpha-diversity, including phylogenetic diversity, the Shannon index for biodiversity and observed OTUs (**Figure 1**). All measures indicate a significantly higher microbiota diversity in the soil compared to the rhizosphere and root microhabitats for both *V. vinifera* 1103P and *V. vinifera* 5BB (Kruskal-Wallis test, *P* ≤ 3 × 10^-6^). Regardless of the rootstock type, the rhizosphere microbiota shows an intermediate diversity between soil and root samples (*P* < 0.05). Principal Coordinates Analysis (PCoA) of weighted and unweighted UniFrac distances reveals a significant segregation among the three different microhabitats analyzed (permutation test with pseudo-F ratios (Adonis), *P* = 0.0001) (**Figure 2**). Interestingly, soil samples show the lowest within-group variability, while greater dispersion is observed for rhizosphere and root samples (Mann-Whitney-Wilcoxon test, false discovery rate (FDR)-adjustment, unweighted UniFrac: soil vs. rhizosphere, *P* = 9 × 10^-19^; soil vs. root, *P* = 6 × 10^-34^; rhizosphere vs. root, *P* = 7 × 10^-32^; weighted UniFrac: soil vs. rhizosphere, *P* = 4 × 10^-3^; soil vs. root, *P* = 4 × 10^-29^; rhizosphere vs. root, *P* = 3 × 10^-18^). According to the taxonomic classification of OTU representative sequences, Proteobacteria, Actinobacteria and Bacteroidetes members dominate all microhabitats (**Figure 3**).

### Differential OTUs Distribution in the Grapevine Root System

**FIGURE 4:**
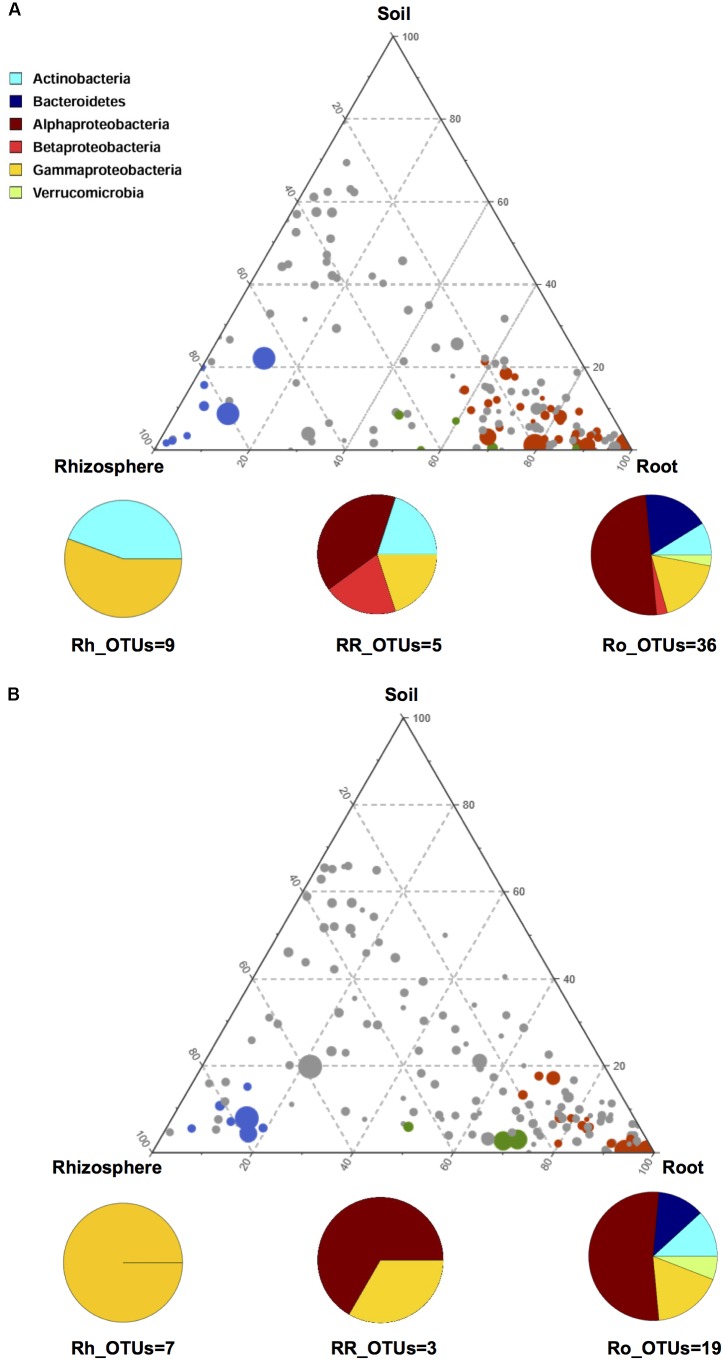
OTU enrichment at the root-soil interface for *V. vinifera* cultivar Lambrusco grafted onto different rootstocks. Ternary plots of all OTUs detected in the dataset with relative abundance >0.5% in at least one sample, in *V. vinifera* 5BB **(A)** and *V. vinifera* 1103P **(B)**. Each circle represents one OTU, and the size is proportional to the weighted relative abundance. Red circles indicate OTUs significantly enriched in the root microhabitat (Ro_OTUs), blue circles indicate OTUs significantly enriched in the rhizosphere microhabitat (Rh_OTUs) and green circles indicate OTUs significantly enriched in both root and rhizosphere microhabitats (RR_OTUs). Mann-Whitney-Wilcoxon test, FDR-adjustment, *P* < 0.05. See also **Supplementary Table S2**.

In order to gain further insights into the recruitment cues of the microbiota thriving at the root-soil interface, we employed a linear analysis of OTUs distribution in soil, rhizosphere and root samples, as previously described (Bulgarelli et al., 2015). Results from this analysis were used to build a ternary plot, highlighting the OTUs showing a specific propensity toward the three microhabitats at the root-soil interface. This approach allowed the identification of three distinct groups of OTUs, each characterized by a specific tropism for (i.e., enriched in) the rhizosphere, the root or both, compared to the other microhabitats (**Figure 4** and **Supplementary Table S2**). The first of these tropic groups, defined as root (Ro)_OTUs, is characterized by all the OTUs showing a preference for (i.e., significantly more abundant in) the root ecosystem, compared to the rhizosphere and the soil (Mann-Whitney-Wilcoxon test, FDR-adjustment, *P* < 0.05). The second tropic group, defined as root/rhizosphere (RR)_OTUs, is composed of OTUs with a tropism for both root and rhizosphere (i.e., enriched in both plant compartments, compared to the soil; *P* < 0.05). The last tropic group, named rhizosphere (Rh)_OTUs, includes all the OTUs more abundant in the rhizosphere ecosystem than in the soil or in the root (*P* < 0.05). According to our data, the Ro_OTUs group is enriched in Proteobacteria, Bacteroidetes and Actinobacteria, and shows a higher number of community-specific OTUs compared to the other groups. On the other hand, the RR_OTUs and Rh_OTUs groups are exclusively dominated by Proteobacteria and Actinobacteria. Interestingly, *V. vinifera* 5BB and *V. vinifera* 1103P show a different OTU compositional layout within the three tropic groups. In particular, members of Betaproteobacteria are included in the Ro_OTUs group of *V. vinifera* 5BB only. Analogously, the Rh_OTUs group only includes Gammaproteobacteria for *V. vinifera* 1103P, while also Actinobacteria for *V. vinifera* 5BB. Finally, the RR_OTUs group of *V. vinifera* 5BB includes bacteria assigned to Alphaproteobacteria, Betaproteobacteria, Gammaproteobacteria and Actinobacteria, while the corresponding group of *V. vinifera* 1103P only contains OTUs belonging to Alphaproteobacteria and Gammaproteobacteria. Strikingly, Ro_OTUs, RR_OTUs and Rh_OTUs groups from 5BB include several putative KSMs that are missing in the corresponding tropic groups from 1103P. In particular, members of *Micrococcaceae* (Rh_OTUs), *Comamonadaceae* (Ro_OTUs and RR_OTUs), *Cytophagaceae* (RR_OTUs), *Sphingomonadaceae*, *Rhizobiaceae*, *Xanthomonadaceae*, and *Microbacteriaceae* (Ro_OTUs) are among the KSMs exclusively present in 5BB (Diep and Hieu, 2013; Zarjani et al., 2013; Zhang and Kong, 2014). Consistently, when comparing the total load of KSMs between the two groups of plants analyzed, 5BB shows a significantly higher relative abundance of KSMs in the Ro_OTUs group compared to 1103P (mean ± SEM, 5BB vs. 1103P, 6.3 ± 1.4% vs. 0.2 ± 0.1%, Mann-Whitney-Wilcoxon test, *P* < 0.05). In addition, 5BB shows a higher number of OTUs assigned to KSMs compared to 1103P (**Supplementary Figure S2**).

### Niche Differentiation of Microorganisms at the Root-Soil Interface

**FIGURE 5:**
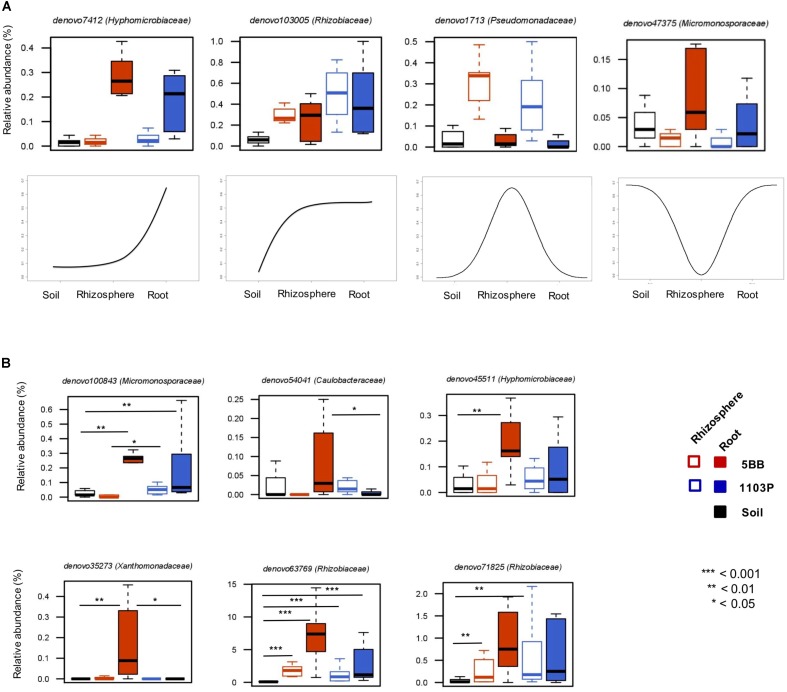
Dynamic plots based on the relative abundance of OTUs with different preference for soil, rhizosphere, and root compartments of *V. vinifera* cultivar Lambrusco grafted onto different rootstocks. **(A)** Examples of OTU niche differentiation across the three microhabitats. **(B)** Differences between the two groups of plants (grafted on the 5BB or 1103P rootstock) in terms of OTU niche preference. *P*-values were assessed by Mann-Whitney-Wilcoxon test. See also **Supplementary Table S2**.

The assessment of the relative preferences of individual OTUs for the different niches in the root system allowed us to define four groups of microorganisms differentially represented at the root-soil interface (**Figure 5A**). The first one is characterized by OTUs (e.g., belonging to the *Hyphomicrobiaceae* family) with low relative abundance in the soil and in the rhizosphere, but highly represented in roots. A second group includes OTUs (e.g., from *Rhizobiaceae*) more abundant in the rhizosphere and in the root, than in the soil. The third comprises OTUs that show a higher percentage in the rhizosphere, compared to the root or the soil, such as members of the *Pseudomonadaceae* family. The last identified group is related to OTUs more represented in the soil and in the root, rather than in the rhizosphere compartment, including *Micromonosporaceae* members.

Next, we specifically explored the OTU niche differentiation by tropic group (Ro_OTUs; RR_OTUs; Rh_OTUs) (**Figure 5B**). Interestingly, according to our data, the same OTUs, even if sharing the same tropism, show different niche preferences based on the rootstock type. In particular, only in *V. vinifera* 5BB plants, 6 OTUs belonging to the *Micromonosporaceae*,*Caulobacteraceae*, *Hyphomicrobiaceae, Xanthomonadaceae*, and *Rhizobiaceae* families, show a higher relative abundance in the root compartment than in the soil and in the rhizosphere, suggesting the relevance of the root environment as a determinant of the bacterial niche preference at the root-soil interface.

## Discussion

By means of next-generation sequencing of the 16S rRNA gene, here we profiled the compositional structure of the soil, rhizosphere and root endosphere microbiota from *V. vinifera* cultivar Lambrusco grafted on two different rootstock hybrids: PAULSEN 1103 (1103P) and KOBER 5BB (5BB). Even though both rootstocks are largely used for grafting Lambrusco cultivars, the 1103P group is prone to develop K absorption problems, with the consequent appearance of atypical red spots on the leaves and a reduction in grape production (Wolpert et al., 2005).

Our data highlight an overall different compositional layout among the three microbial ecosystems at the root-soil interface: soil, rhizosphere and root endosphere. Interestingly, we observed a progressive decrease of microbial diversity from soil to rhizosphere and root endosphere. Paralleled by a corresponding increase in individual specificity, this behavior reflects the typical organizing principle of holobionts microbiomes (Compant et al., 2010; Liu et al., 2017; Thompson et al., 2017). Thus, our data suggest that the enrichment in Actinobacteria, Bacteroidetes and Proteobacteria observed in the root endosphere-inhabiting microbiota is the result of a gated community assembled from the, taxonomically congruent, surrounding soil and rhizosphere biomes.

Through a ternary plot analysis, we were successful in discriminating microorganisms showing a specific tropic behavior for the plant ecosystems (i.e., rhizosphere and root endosphere), compared to the soil. Interestingly, the root endosphere-tropic microbial group is composed of the largest number of microhabitat-characteristic OTUs, showing higher taxonomic diversity compared to the other two tropic groups. Strikingly, differences in specific tropisms for the plant ecosystems emerge from the comparison between the two rootstocks, with 1103P being depleted in several OTUs some of which belonging to KSMs, which are instead detected in 5BB. For instance, OTUs assigned to *Micrococcaceae* are represented only in the 5BB rhizosphere. However, the most severe depletion of KSMs-assigned OTUs is observed in the root endosphere of 1103P, with the absence of several well-known KSMs, including *Cytophagaceae*, *Rhizobiaceae, Xanthomonadaceae* and *Comamonadaceae* members (Diep and Hieu, 2013; Zarjani et al., 2013; Zhang and Kong, 2014), which are detected only in the 5BB root endosphere. This overall decrease in KSM diversity in the 1103P plant-associated microbiomes could account for the problems of K absorption characteristic of *V. vinifera* cultivar Lambrusco grafted on the 1103P rootstock. Indeed, the lack of KSMs – capable of easily metabolizing insoluble K into soluble forms, making it available to the plant – can compromise K absorption by the plant, leading to poor development of roots, slow growth and increased susceptibility to diseases and pest (Keller, 2010; Etesami et al., 2017). According to our findings, the two rootstocks differently modulate the niche preference of microorganisms at the root-soil interface. For instance, several microbes, belonging to the families *Micromonosporaceae, Caulobacteraceae*, *Hyphomicrobiaceae*, *Xanthomonadaceae*, and *Rhizobiaceae*, the latter two including KSMs, show only in 5BB plants a higher relative abundance in the root endosphere than in the soil and in the rhizosphere. Supposedly, these microorganisms are selected from the plant at the root interface as a result of the plant-bacteria cross-talk mediated by the rhizodeposition of metabolites (Bulgarelli et al., 2013). Growing up inside the root tissues, these microorganisms may provide a number of beneficial functions for the plant, such as indirect pathogen protection, phosphorus solubilization and nitrogen metabolism (Bulgarelli et al., 2013).

Taken together, our data indicate that different rootstocks result into different plant-associated microbiomes, as a result of different plant-bacteria interaction processes at the root-soil interface. In particular, compared to 1103P, the 5BB plants select for a higher diversity of KSMs in both the rhizosphere and root endosphere compartments, possibly explaining the inadequate K absorption observed in the 1103P group. Besides confirming the importance of the rootstock as a determinant of the composition of plant microbiomes (rhizosphere and root endosphere) (Samad et al., 2017; Marasco et al., 2018), our findings suggest the importance of rootstock-selected plant microbiomes as possible regulators of K absorption by *V. vinifera*.

In conclusion, different rootstocks used for grafting the same cultivar (i.e., the cultivar Lambrusco) select for different plant microbiotas in root endosphere and rhizosphere compartments. This process has strong repercussions in terms of plant physiology and health, such as the regulation of K absorption. Our results can provide the basis for future applications (e.g., microbial inocula) aimed at favoring a better microbiome composition in *V. vinifera* cultivar Lambrusco grafted onto 1103P, counteracting nutrient absorption deficiency when rootstock replacement is not possible. Further studies are needed to isolate and functionally characterize KSMs from the rhizosphere and root ecosystems, as well as to provide direct biological evidence of their impact on the rootstock performance in K absorption. Different K applications could be instrumental to unravel the mechanisms by which plant microbiomes regulate the plant nutrient status and its growth. Moreover, chemical mediators orchestrating the microbes-host cross-talk at the root-soil interface and eventually favoring the recruitment of KSMs, need to be discovered, opening the perspective to a knowledge-based chemical modulation of plant microbiomes to sustain the establishment of configurations promoting plant health and hence its productivity.

## Author Contributions

DV and AB carried out the field work. ST performed the DNA extraction and library preparation. FD run the sequencing. FD and SR performed the bioinformatics analysis. SR, ST, and MC designed the study. FD, SR, MC, and ST wrote the manuscript. EB and PB participated in the data interpretation and commented on the draft. All authors read and approved the final manuscript.

## Conflict of Interest Statement

The authors declare that the research was conducted in the absence of any commercial or financial relationships that could be construed as a potential conflict of interest.

## Footnotes

1 https://www4.istat.it/it/archivio/207188

2 https://www.mg-rast.org/mgmain.html?mgpage=token&token=rcSlcBTSY85CpNpN7552xeEBUUZYonG3TFqXfrYHH_f4ACrVPu

3 https://cran.r-project.org/web/packages/vegan/index.html

## Supplementary Material

FIGURE S1 Location of experimental fields and leaves of *V. vinifera* cultivar Lambrusco with impaired potassium absorption. **(A)** Map of Italy and specifically of Emilia-Romagna region. The red dot localizes the experimental fields in Finale Emilia (Modena). **(B)** Images showing the leaves of *V. vinifera* cultivar Lambrusco grafted on the rootstock hybrid *V. berlandieri* × *V. rupestris* PAULSEN 1103, with the typical red spots caused by potassium deficiency.

FIGURE S2 Number of OTUs assigned to potassium-solubilizing microorganisms (KSMs) in the root-tropic group (Ro_OTUs) for both 5BB and 1103P plant groups.

TABLE S1 Physical and chemical characterization of the experimental soil substrates used in the study.

TABLE S2 OTUs characterizing the root, the rhizosphere or both compartments for *V. vinifera* cultivar Lambrusco grafted onto the rootstock 5BB or 1103P. Ro, root; Rh, rhizosphere; RR, root/rhizosphere.
