# Supplementary figures and images for: The Rootstock Regulates Microbiome Diversity in Root and Rhizosphere Compartments of Vitis vinifera Cultivar Lambrusco
Source: Front Microbiol. 2018 Sep 26;9:2240. doi: 10.3389/fmicb.2018.02240 (PMC6169447; doi:10.3389/fmicb.2018.02240)

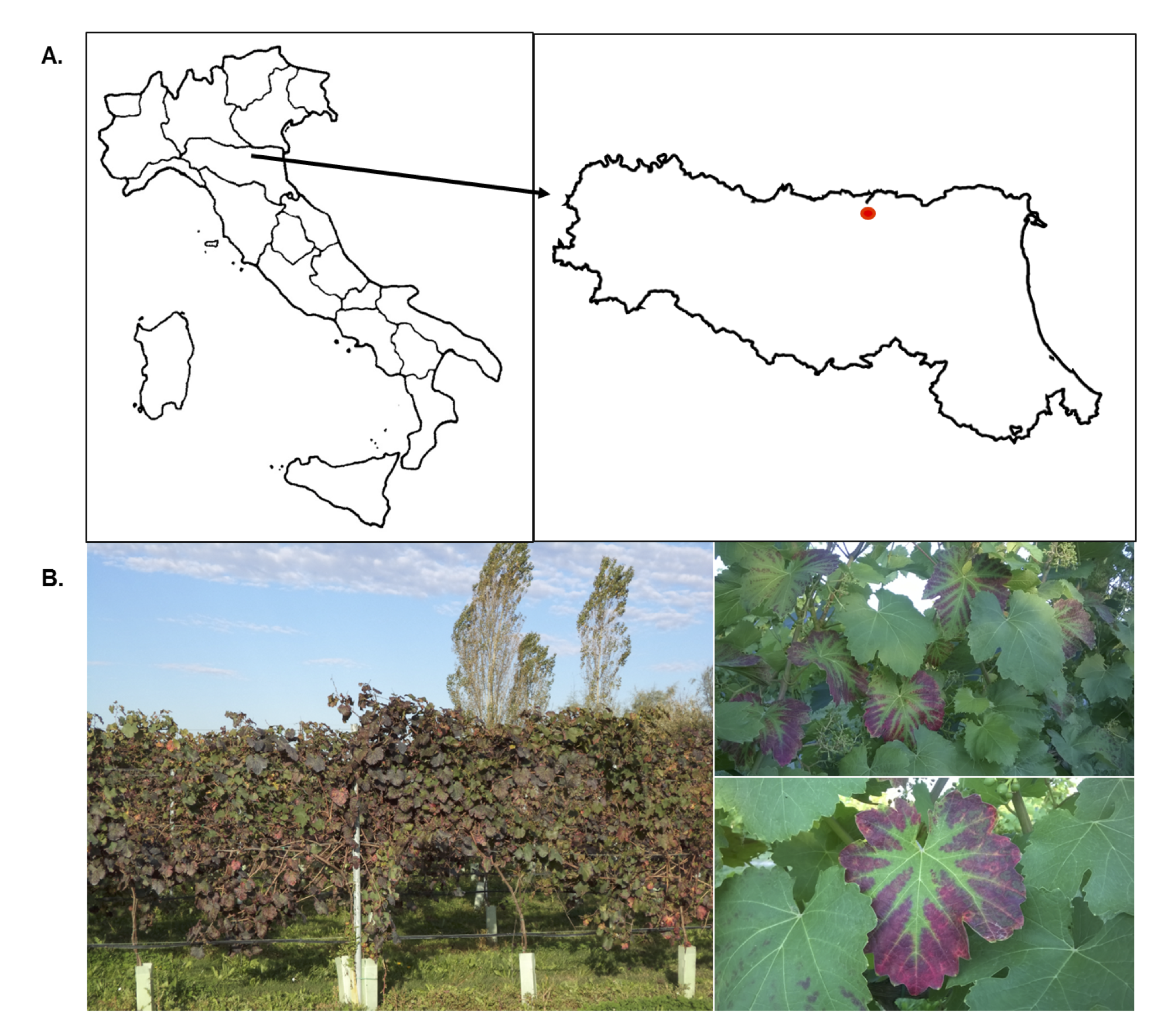

Supplement: FIGURE S1 — Location of experimental fields and leaves of V. vinifera cultivar Lambrusco with impaired potassium absorption. (A) Map of Italy and specifically of Emilia-Romagna region. The red dot localizes the experimental fields in Finale Emilia (Modena). (B) Images showing the leaves of V. vinifera cultivar Lambrusco grafted on the rootstock hybrid V. berlandieri × V. rupestris PAULSEN 1103, with the typical red spots caused by potassium deficiency. [file Image_1.TIFF]

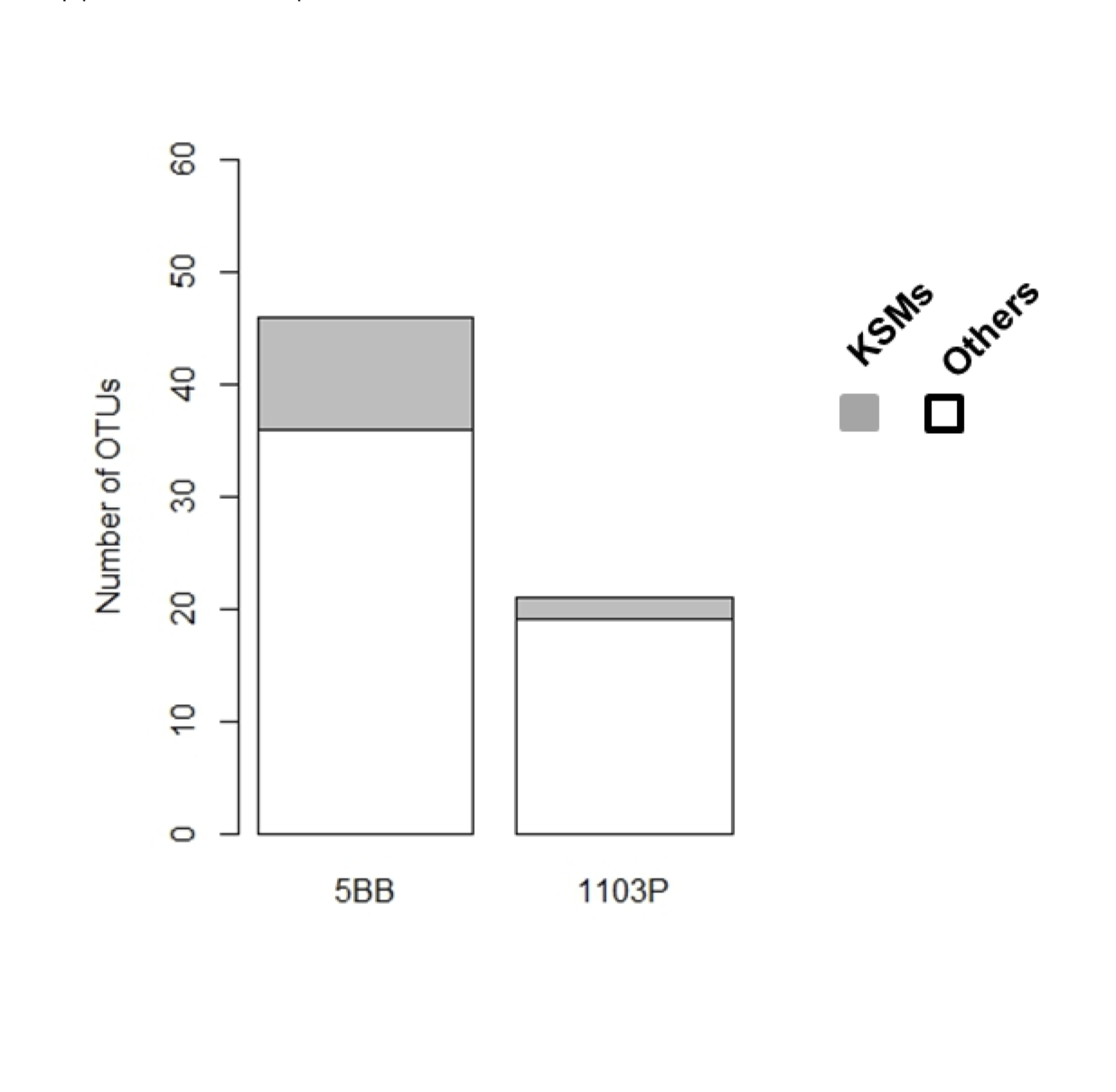

Supplement: FIGURE S2 — Number of OTUs assigned to potassium-solubilizing microorganisms (KSMs) in the root-tropic group (Ro_OTUs) for both 5BB and 1103P plant groups. [file Image_2.TIFF]
