# Supplementary material for: The Rootstock Regulates Microbiome Diversity in Root and Rhizosphere Compartments of Vitis vinifera Cultivar Lambrusco
Source: Front Microbiol. 2018 Sep 26;9:2240. doi: 10.3389/fmicb.2018.02240 (PMC6169447; doi:10.3389/fmicb.2018.02240)
Supplement: TABLE S1 — Physical and chemical characterization of the experimental soil substrates used in the study. [file Table_1.docx]

**Supplementary Table S1.** **Physical and chemical characterization of the experimental soil substrates used in the study.**

| **TEXTURE** | **Sampling date: Nov 9, 2016**  **Value** |
| --- | --- |
| Sand | 266 g/kg |
| Silt | 518 g/kg |
| Clay | 216 g/kg |
| Total Limestone | 202 g/kg |
| Active Limestone | 40 g/kg |
| Organic Substance | 14.2 g/kg |
| pH in H_2_O | 8 |
|  |  |
| **MINERAL CONTENT** |  |
| Total Nitrogen (N) | 0.8 g/kg |
| Phosphorous (P) | 12 ppm |
| Potassium (K) | 145 ppm |
| CaO | 4011 ppm |
| Ca^2+^ | 2848 ppm |
| MgO | 148 ppm |
| Mg^2+^ | 88 ppm |
| Carbon/Nitrogen | 10.2 |
